# Functional network reorganization following VIM-MRgFUS for essential tremor

**DOI:** 10.1016/j.neurot.2026.e00864

**Published:** 2026-02-27

**Authors:** Jinlong Liu, Jonas Krauss, Veronika Purrer, Valeri Borger, Markus Essler, Alexander Radbruch, Ullrich Wüllner, Neeraj Upadhyay, Henning Boecker

**Affiliations:** a University of Bonn, University Hospital Bonn, Clinical Functional Imaging Group, Department of Nuclear Medicine, Bonn, Germany; b German Center for Neurodegenerative Diseases (DZNE), Bonn, Germany; c University of Bonn, University Hospital Bonn, Clinic for Parkinson's Disease, Sleep Disorders, and Movement Disorders, Bonn, Germany; d University of Bonn, University Hospital Bonn, Department of Neurosurgery, Bonn, Germany; e University of Bonn, University Hospital Bonn, Department of Nuclear Medicine, Bonn, Germany; f University of Bonn, University Hospital Bonn, Department of Neuroradiology, Bonn, Germany

**Keywords:** Essential tremor, MRgFUS, Brain networks, Graph theory analysis, Ventral intermediate nucleus

## Abstract

Magnetic resonance-guided focused ultrasound (MRgFUS) is a promising, noninvasive therapeutic approach for essential tremor (ET), yet the effects of the respective lesions on functional brain network organization remain poorly understood. Here, we performed graph theory analysis to investigate changes in small-world properties and modular architecture in ET patients six months after unilateral MRgFUS of the thalamic ventral intermediate (VIM) nucleus. Small-worldness and normalized clustering coefficient increased significantly after MRgFUS, while clustering coefficient, characteristic path length, normalized characteristic path length, global efficiency, and local efficiency remained unchanged. Modular organization was largely preserved, but trend-level enhancements in inter-modular connectivity were observed between frontoparietal and subcortical modules, as well as between frontoparietal and frontotemporal-parietal modules. Within these modules, betweenness centrality increased significantly in specific cortical hubs, including the left superior frontal gyrus, right superior parietal lobule, and left postcentral gyrus. These findings indicate that unilateral VIM-MRgFUS induces selective functional network reorganization, particularly affecting relative clustering and nodal centrality patterns.

## Introduction

Essential tremor (ET) is the most common movement disorder, characterized by bilateral postural or kinetic tremor, often affecting the upper limbs [1]. The prevalence of ET rises with advancing age, affecting approximately 5 % of individuals over 65 years [2]. Although pharmacological therapies remain the first-line treatment, many patients exhibit poor responses, leading to substantial functional disability and socioeconomic burden [3,4]. Surgical options such as stereotactic thalamotomy and deep brain stimulation (DBS) are available, but many patients refuse due to the invasiveness of these procedures. Magnetic resonance-guided focused ultrasound (MRgFUS) has emerged as a promising noninvasive treatment for patients with medication-refractory ET [5]. This incisionless technique provides real-time temperature monitoring without the need for general anesthesia or ionizing radiation [6]. Long-term follow-up studies report durable tremor suppression up to five years after unilateral MRgFUS thalamotomy, accompanied by sustained improvements in quality-of-life measures and without evidence of progressive or delayed complications [7,8].

The prevailing pathophysiological hypothesis proposes that ET arises from abnormal oscillatory activity within the cerebellum and the cerebello-thalamo-cortical (CTC) circuit, often referred to as the “tremor network” [9]. Increasing evidence suggests that ET involves widespread brain network dysfunction, extending beyond the classical CTC network [10,11]. We therefore adopted network analysis to investigate the therapeutic mechanisms of MRgFUS in ET patients.

Graph theory analysis offers a robust mathematical framework to characterize the topological properties of brain networks, modeling brain regions as nodes and their functional or structural connections as edges [12]. A key concept is the small-world organization, which reflects an optimal balance between local specialization (clustering coefficient) and global integration (characteristic path length) [13]. Loss of small-world properties, including reduced clustering coefficient, small-worldness, and increased characteristic path length, has been reported in ET [11,14,15]. Another fundamental property, modularity, quantifies the extent to which a network can be partitioned into functionally specialized communities and provides a template for understanding reorganization of large-scale brain networks [16,17].

Several recent studies have applied graph theory analysis to investigate the effects of MRgFUS on brain networks in ET. Early work suggested that MRgFUS regulates interactions over the motor network via symptom-related connectivity changes but accompanies transient, symptom-unrelated diaschisis in the global brain network [18]. Structural network analyses have demonstrated increased clustering coefficient and rich-club connectivity alongside reduced characteristic path length after MRgFUS [19]. More recently, an fMRI study reported that MRgFUS thalamotomy promoted corticostriatal connectivity activation associated with tremor improvement in ET patients [20]. Despite these advances, the impact of MRgFUS on functional network topology—particularly small-world properties and modular organization—remains poorly understood.

Given prior evidence of disrupted small-world architecture and decreased network efficiency in ET [11,14,15] and our own findings of a shift toward a more integrated functional state following MRgFUS [21], we hypothesize that MRgFUS promotes a shift toward a more efficient network configuration in ET patients. Specifically, we hypothesize that MRgFUS improves small-world characteristics and network efficiency, and reduces network segregation.

## Materials and Methods

### Patients

Patients aged ≥18 years with a diagnosis of definite drug-refractory ET [22] were enrolled at the University Hospital Bonn, Germany. Tremor was present bilaterally in all patients. The inclusion criteria for MRgFUS are described in detail in the German Clinical Trials Registry (DRKS00016695). All patients underwent unilateral VIM-MRgFUS between 2019 and 2024.

Written informed consent was obtained from all patients after a detailed explanation of the study aims and possible risks. The study was approved by the local ethics committee of the University Hospital Bonn (No. 314/18) and was conducted in accordance with the Declaration of Helsinki.

### Clinical assessment

We assessed the tremor severity using the Fahn-Tolosa-Marin Clinical Rating Scale for Tremor (FTM) [23] before and 6 months after MRgFUS. The FTM scale comprises three parts (A, B, and C), each assessing a distinct aspect of tremor severity. Part A measures tremor at rest, during postural holding, and during action, scored separately for the left and right side. Part B evaluates action tremor of the upper limbs through tasks such as drawing and writing. Finally, part C captures the patient's subjective perception of tremor-related disability in daily activities, including dressing, drinking, and speaking. We combined parts A and B into a modified FTM A/B including only hand-related items to avoid data skewing caused by deviating presence of leg, head or voice tremor. Scores were calculated separately for the treated and untreated side.

### MRgFUS intervention

Before MRgFUS, a preoperative CT was performed to assess contraindications and determine a skull density ratio of ≥0.3. The location of the lesion within the VIM was determined according to the established indirect targeting procedure using standard atlas-based stereotactic approximations [24]. Diffusion tensor imaging (DTI) allowed visualizing specifically the dentato-thalamo-cortical tract (DTCT) while avoiding the corticospinal and the medial lemniscus tracts. Before the final lesion was placed, subthreshold sonications (<50 °C) were performed, allowing a neurologist to identify targets with the best tremor reduction and absence of adverse effects. The final lesions were induced by thermal ablation via MRgFUS (ExAblate Neuro 4000, Insightec) using, on average, 4.5 ± 1.5 sonications >55 °C**.** The details of the MRgFUS procedure have been described previously [25,26].

### MRI procedures

#### Acquisition

ET patients underwent MRI examinations at baseline and 6-month follow-up at the University Hospital Bonn. A 3D T1-weighted magnetization-prepared rapid gradient echo (MPRAGE) sequence was used to acquire high-resolution structural images with the following parameters: matrix size: 256× 256 mm; voxel size: 1 × 1 × 1 mm^3^; echo time (TE): 3.93 ms; repetition time (TR): 7.293 ms; flip angle (FA): 15°; scan duration: 4:39 min. Resting state (Rs)-fMRI images were acquired as follows: matrix size: 64 × 64 mm; voxel size: 3.59 × 3.59 × 3.59 mm^3^; TE: 5.5001 ms; TR: 2.595 s; FA: 90°; slice order: interleaved (first odd, then even); 250 rs-fMRI images per scan; total scan duration: 11 min. During rs-fMRI acquisition, participants were asked to remain still, close their eyes, stay awake, and refrain from deliberate mental activity.

### Preprocessing

MRI data preprocessing was carried out using CONN toolbox version 22a (http://www.nitrc.org/projects/conn) in combination with SPM12 (https://www.fil.ion.ucl.ac.uk/spm/software/spm12/), implemented in MATLAB R2023b. Before analysis, data from patients treated in the right VIM were R-L flipped to ensure consistency across hemispheres. The preprocessing pipeline included the following steps: (1) functional data were realigned and unwarped using fieldmaps for susceptibility distortion correction; (2) temporal misalignment was corrected following the SPM slice-timing correction (STC) procedure; (3) potential outlier scans were identified using ART as acquisitions with framewise displacement above 0.9 mm or global BOLD signal changes above 5 standard deviations [27]; (4) functional and anatomical images were coregistered and normalized into MNI space, segmented into gray matter, white matter, and cerebrospinal fluid (CSF), and resampled to 2 mm isotropic voxels using an indirect normalization procedure; (5) functional data were smoothed using spatial convolution with a Gaussian kernel of 6 mm full width half maximum (FWHM); (6) denoising was performed by regressing out confounds, including five CompCor components from white matter, five from CSF, 12 motion parameters (six realignment parameters and their first derivatives), identified outlier scans, and linear trends, followed by temporal high-pass filtering above 0.01 Hz.

### Network construction

To construct the functional connectivity matrix, we used the Human Brainnetome Atlas (https://atlas.brainnetome.org/), which was resampled to 2 mm isotropic voxels. This parcellation yielded 273 regions of interest (ROIs), including 246 cerebral and 27 cerebellar regions. For each ROI, the representative time series was extracted as the mean BOLD signal across all voxels in that region. Pairwise functional connectivity was then estimated using Pearson's correlation coefficients between the time series of all ROI pairs, yielding individual correlation matrices. To improve the normality of the correlation values, the correlation coefficients were transformed using Fisher's r-to-z transformation. In line with previous studies and considering the ongoing debate regarding the physiological interpretation of negative correlations [28,29], all negative correlation values were set to zero prior to further analyses.

To investigate the topological properties of functional brain networks while reducing the bias introduced by single-threshold selection, we applied a sparsity threshold range from 0.05 to 0.50 in increments of 0.01. To ensure the appropriateness of this range, two criteria were verified for all participants: (a) the average degree of the network exceeded 2 × log(N), where N denotes the number of nodes, and (b) network small-worldness was preserved, defined as σ > 1.1 across all thresholds [13,30]. Weighted functional connectivity matrices were subsequently converted into binary adjacency matrices according to the predefined sparsity thresholds: connections with correlation coefficients above the threshold were assigned a value of 1, whereas those below the threshold were set to 0.

## Graph theory analysis

Graph theory analysis was conducted using the Graph Theoretical Network Analysis toolbox (GRETNA) (https://www.nitrc.org/projects/gretna). For each threshold, both global and local metrics were analyzed. Global metrics, including the clustering coefficient (Cp), characteristic path length (Lp), normalized clustering coefficient (γ), normalized characteristic path length (λ), small-worldness (σ = γ/λ), global efficiency (Eg), local efficiency (Eloc), and modularity (Q) were calculated. Local metrics comprised degree centrality, nodal efficiency, and betweenness centrality. Detailed descriptions of the computation and interpretation of these metrics can be found in previously published literature [31]. To minimize the bias introduced by selecting a single sparsity threshold, we further adopted the area under the curve (AUC) approach. This method integrates the values of each network metric across a predefined sparsity range, thereby providing a more robust and summarized index for network characterization. Compared with single-threshold analysis, the AUC metric reduces dependence on arbitrary threshold selection and has been used in previous studies [30,32].

### Modular analysis

Given the central role of modularity in brain network organization, we further conducted modular analysis to characterize community structure. Modularity reflects the extent to which a network can be subdivided into communities, and the maximum modularity value Q quantifies how well a partition segregates tightly connected nodes into distinct modules [16]. To examine modular architecture at the population level, we first constructed group-level brain networks by averaging all individual functional connectivity matrices at baseline and binarizing the group-mean matrix using a sparsity threshold of 0.15, which represented the sparsest density with minimal probability of spurious connections [33]. In previous studies, a modularity value of Q ≥ 0.3 has generally been accepted as indicative of a non-random community structure [34]. Community detection was performed with the Brain Connectivity Toolbox [31], employing a modified greedy optimization algorithm [35] to identify the optimal partition. After obtaining the modular structure, we examined the distribution of inter- and intra-modular connections to reveal the connectivity profiles across different brain regions. To further explore potential drivers of altered inter- and intra-modular interactions, nodal topological measures were additionally assessed based on inter- and intra-modular connectivity changes. In addition to the nodal metrics described above, we also calculated the participation coefficient, which quantifies the extent to which a node maintains inter-modular communication by distributing its links across multiple modules [31].

### Statistical analysis

All statistical analyses were performed using SPSS version 27.0 (IBM Corporation, USA). The Shapiro-Wilk test was applied to assess the normality of the data. For comparisons between baseline and six months after MRgFUS, paired t-tests were used for normally distributed data, whereas the Wilcoxon signed-rank test was applied for non-normally distributed data. Correlations between topological properties and FTM scores were examined using the nonparametric Spearman rank correlation test. Results with *p* < 0.05 were regarded as statistically significant. Given our a priori hypothesis that MRgFUS will improve small-world characteristics and network efficiency and reduce network segregation, one-tailed tests were applied for global metrics. For all other comparisons, two-tailed tests were used. Multiple comparisons were addressed by applying false discovery rate (FDR) correction [36] to local metrics as well as modular analyses, including inter- and intra-modular interactions and nodal properties. Global metrics were analyzed without correction [37,38].

## Results

### Demographic and clinical data

A total of 46 patients diagnosed with drug-refractory ET underwent unilateral VIM-MRgFUS. Of these, nine patients were excluded due to insufficient image quality, resulting in a final cohort of 37 patients (23 males and 14 females). The mean age at the time of treatment was 69.71 ± 10.81 years (mean ± standard deviation). The average age of onset and disease duration were 42.53 ± 22.69 years and 29.03 ± 18.23 years, respectively. Among the included patients, 35 (94.59 %) were right-handed, 18 (48.65 %) demonstrated alcohol sensitivity, and 32 (86.49 %) underwent left-sided VIM-MRgFUS. Demographic details are provided in Table 1**.**

**Table 1:** Demographical data of ET patients.

Characteristics	ET Patients (n = 37)
Age	69.71 ± 10.81
Sex: Male/female	23/14
Handedness: Right/non-right-handed	35/2
Age of onset	42.53 ± 22.69
Disease duration	29.03 ± 18.23
Alcohol sensitivity: Yes/no	18/19
Treatment location VIM: Left/right	32/5

**Fig. 1:**
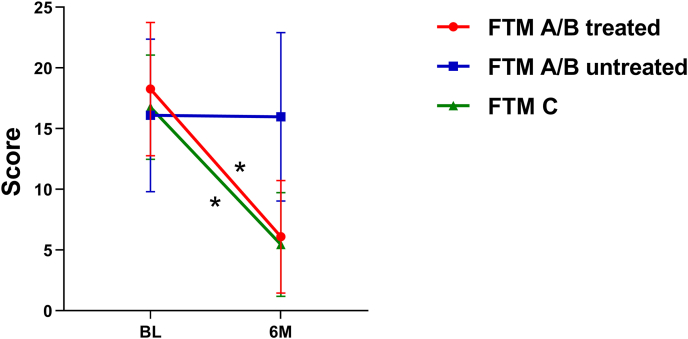
FTM score changes between baseline (BL) and 6-month (6 M) follow-up after MRgFUS. Significant differences are marked with an asterisk (∗*p* < 0.001). Error bars represent standard deviations.

The FTM tremor scores demonstrated a significant reduction on the side contralateral to the treated VIM, decreasing from 18.31 ± 5.55 (mean ± standard deviation) at baseline to 6.17 ± 4.66 at 6 months post-MRgFUS (t = 12.234, *p* < 0.001), corresponding to a mean improvement of 65.39 %. In contrast, the non-treated side showed no significant change, with scores of 16.14 ± 6.35 at baseline and 15.94 ± 7.03 at 6 months (t = 0.322, *p* = 0.750). Furthermore, the FTM C improved significantly, decreasing from 16.54 ± 4.20 at baseline to 5.61 ± 4.44 at 6 months (t = 12.457, *p* < 0.001), representing a mean improvement of 66.97 % (Fig. 1).

## Alterations of global and nodal topological properties

**Fig. 2:**
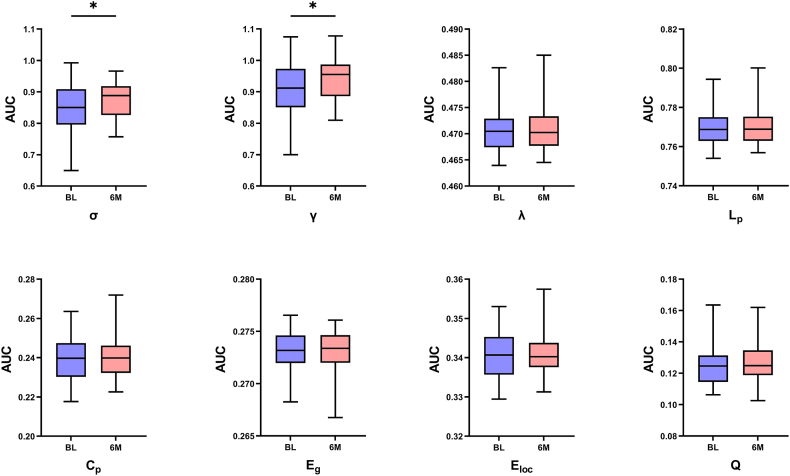
Alterations in the AUC value of global topological properties between baseline and 6 months. Significant differences are marked with an asterisk (∗*p* < 0.05). Abbreviations: AUC, area under the curve; σ, small-worldness; γ, normalized clustering coefficient; λ, normalized characteristic path length; Cp, clustering coefficient; Lp, characteristic path length; Eloc, local efficiency; Eg, global efficiency; Q, modularity.

The average degree at a threshold of 0.05 was 12.61 (>2 × log 273 = 11.22), confirming the adequacy of network density. Brain networks at both baseline and 6 months post-MRgFUS exhibited small-world organization (σ > 1.1) across all preselected thresholds (0.05–0.5, step = 0.01), supporting the use of this threshold range for subsequent analyses. Global topological analysis demonstrated significant increases in the AUC of both σ (baseline: 0.85 ± 0.08 vs. 6 months: 0.88 ± 0.06, *p* < 0.05) and γ (baseline: 0.92 ± 0.09 vs. 6 months: 0.94 ± 0.07, *p* < 0.05) following MRgFUS (Fig. 2). In contrast, the AUC of λ, Cp, Lp, Eg, Eloc or Q showed no significant change at 6 months compared with baseline (all *p* > 0.05; Fig. 2).

Nodal topological analysis showed no significant differences in the AUC of degree centrality, nodal efficiency, or betweenness centrality across all brain regions at 6 months post-MRgFUS compared with baseline (all *p* > 0.05, FDR-corrected; detailed nodal results are provided in Supplementary Table 1).

### Modular organization

**Fig. 3:**
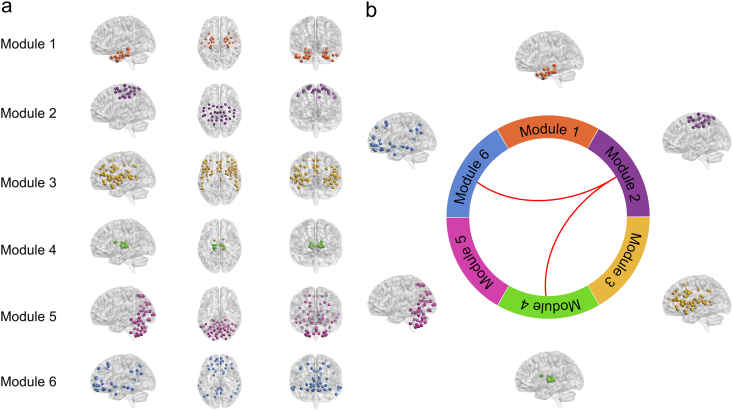
Modular organization of brain networks. (a) Six identified modules are shown with their corresponding ROIs in sagittal, axial, and coronal views. (b) A schematic circular diagram illustrates the six modules, with red lines suggesting a trend toward enhanced inter-modular interaction.

Community detection analysis at the threshold of 0.15 revealed a modular organization consisting of six functional modules (Q = 0.430). Specifically, the modules included: the temporal module (module 1, 31 ROIs), the frontoparietal module (module 2, 33 ROIs), the frontotemporal-insular module (module 3, 77 ROIs), the subcortical module (module 4, 18 ROIs), the parieto-occipital-cerebellar module (module 5, 69 ROIs), and the frontotemporal-parietal module (module 6, 45 ROIs) (Fig. 3a).

### Inter- and intra-modular connectivity

No significant changes were observed in either inter- or intra-modular functional connectivity among the six detected modules at 6 months compared with baseline (all *p* > 0.05, FDR-corrected; Table 2, Table 3). However, a trend toward increased connectivity was observed between module 2 and module 4, as well as between module 2 and module 6 at 6 months (*p* < 0.05, uncorrected) (Fig. 3b).

**Table 2:** Inter-modular connectivity.

	BL (mean ± SD)	6 M (mean ± SD)	*p* (uncorr.)	*p* (FDR)
module1&2	24.11 ± 27.19	22.7 ± 18.23	0.773	0.804
module1&3	158.97 ± 77.74	143.38 ± 91.71	0.318	0.795
module1&4	29.70 ± 25.63	35.16 ± 33.23	0.417	0.804
module1&5	193.59 ± 79.94	214.14 ± 102.47	0.274	0.795
module1&6	177.41 ± 75.32	180.81 ± 58.16	0.804	0.804
module2&3	403.22 ± 137.66	379.89 ± 145.47	0.485	0.804
module2&4	13.05 ± 12.91	19.76 ± 23.61	0.039∗	0.293
module2&5	281.70 ± 136.39	265.00 ± 123.17	0.458	0.804
module2&6	70.95 ± 34.82	91.16 ± 43.38	0.013∗	0.195
module3&4	82.27 ± 56.81	87.30 ± 60.83	0.626	0.804
module3&5	329.24 ± 124.45	365.73 ± 112.11	0.128	0.640
module3&6	466.76 ± 127.79	460.08 ± 100.91	0.738	0.804
module4&5	52.86 ± 41.18	54.70 ± 36.25	0.781	0.804
module4&6	46.73 ± 29.34	48.78 ± 24.70	0.719	0.804
module5&6	310.30 ± 100.05	329.97 ± 98.48	0.309	0.795

Inter-modular functional connectivity before and after MRgFUS treatment is shown. Module pairs with trend-level increases at 6 months are indicated (∗*p* < 0.05, uncorrected). Abbreviations: BL, baseline; 6M, 6 months.

**Table 3:** Intra-modular connectivity.

	BL (mean ± SD)	6 M (mean ± SD)	*p* (uncorr.)	*p* (FDR)
module1	211.05 ± 59.37	211.03 ± 59.12	0.998	0.998
module2	293.00 ± 72.98	311.46 ± 69.05	0.125	0.453
module3	1011.18 ± 201.10	986.11 ± 168.88	0.406	0.609
module4	92.68 ± 18.86	93.92 ± 17.25	0.682	0.818
module5	786.62 ± 161.85	754.86 ± 152.44	0.313	0.609
module6	398.59 ± 72.67	378.03 ± 71.13	0.151	0.453

Intra-modular functional connectivity before and after MRgFUS treatment is shown. Abbreviations: BL, baseline; 6M, 6 months.

### Nodal analysis in modules showing higher inter-modular connectivity

**Fig. 4:**
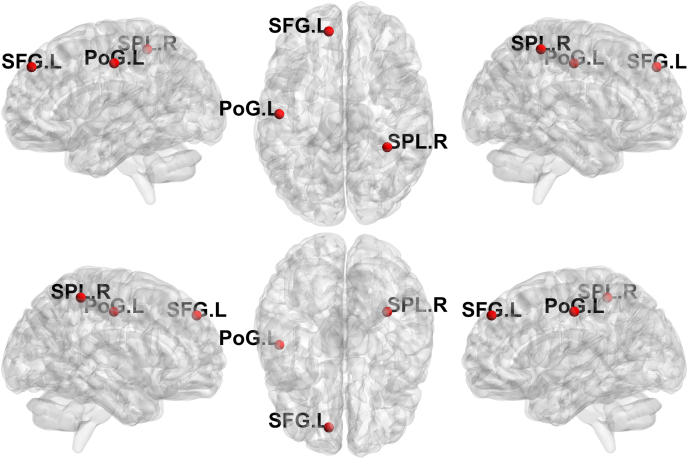
Nodes showing significant changes in betweenness centrality after MRgFUS (*p* < 0.05, FDR-corrected). Abbreviations: SFG.L, left superior frontal gyrus; SPL.R, right superior parietal lobule; PoG.L, left postcentral gyrus. Detailed nodal information is provided in Supplementary Table 2.

Within the modules showing a trend toward higher inter-modular interactions, betweenness centrality increased significantly in three regions: the left superior frontal gyrus (*p* = 0.036, FDR-corrected), the right superior parietal lobule (*p* = 0.036, FDR-corrected), and the left postcentral gyrus (*p* = 0.045, FDR-corrected; see Fig. 4 for detailed locations). In contrast, there were no significant changes in degree centrality, nodal efficiency, or participation coefficient within these modules (all *p* > 0.05, FDR-corrected; detailed nodal information is provided in Supplementary Table 2).

## Correlations among topological properties, modular connectivity, and clinical outcomes

Spearman correlation analysis revealed no significant associations between changes in global topological properties and clinical improvements at 6 months post-MRgFUS. Specifically, changes in σ were not significantly correlated with FTM A/B (r = 0.078, *p* = 0.655) or FTM C (r = 0.235, *p* = 0.228), and changes in γ were not significantly correlated with FTM A/B (r = 0.033, *p* = 0.853) or FTM C (r = 0.221, *p* = 0.259). At the nodal level, no significant correlations were observed between betweenness centrality and clinical scores. However, the betweenness centrality of the right superior parietal lobule was positively correlated with increased inter-modular interaction between module 2 and module 4 (r = 0.432, *p* = 0.008; Table 4).

**Table 4:** Correlation of BC with clinical scores and inter-modular connectivity.

Variable	FTM A/B	FTM C	module 2&4	module 2&6
BC_SFG.L	0.034 (0.848)	0.124 (0.530)	–	0.101 (0.551)
BC_SPL.R	0.110 (0.531)	0.168 (0.392)	**0.432 (0.008)**	0.116 (0.493)
BC_PoG.L	0.001 (0.995)	0.082 (0.680)	0.042 (0.805)	0.250 (0.136)

Values are presented as r (*p*). Significant correlation is indicated in bold. Abbreviations: BC, betweenness centrality; SFG.L, left superior frontal gyrus; SPL.R, right superior parietal lobule; PoG.L, left postcentral gyrus.

## Discussion

We performed graph theory analysis to investigate the impact of MRgFUS on small-world properties and modular organization in ET. Small-worldness and normalized clustering coefficient increased significantly after MRgFUS, whereas clustering coefficient, characteristic path length, normalized characteristic path length, global efficiency, local efficiency, and modularity remained unchanged. Modular organization was largely preserved at six months, but trend-level increases in inter-modular connectivity were observed, notably between frontoparietal and subcortical modules, as well as between frontoparietal and frontotemporal-parietal modules. Within the modules exhibiting higher connectivity, the betweenness centrality of specific cortical hubs increased, indicating a selective strengthening of key nodes within the functional network.

Small-world networks are characterized by high local clustering and short path lengths, enabling both specialized (segregated) processing and efficient global integration [13]. This organization is considered an economical solution that balances wiring cost with rapid information transfer and has been consistently observed in structural and functional brain networks across scales [[39], [40], [41]]. However, neuroimaging studies in ET have reported disrupted global topology, including reduced clustering and efficiency and increased path length, indicating a deviation from optimal small-world balance, with some features consistent with a trend toward network randomization [11,14,15]. In our study, σ and γ significantly increased after MRgFUS, while Cp, Lp, λ, Eg, and Eloc did not show significant changes. Importantly, although Cp remained stable, the increase in γ reflects relative enhancement of clustering compared to random networks, rather than a direct increase in raw clustering. This pattern indicates a subtle topological reconfiguration in which the functional network architecture becomes more small-world-like in a relative sense. In contrast, a structural-network study reported a different yet complementary pattern: Cp, Eg, and Eloc significantly increased and Lp decreased postoperatively, changes that would typically predict higher σ, yet σ did not reach statistical significance [19]. Rather than indicating contradictory findings, these results suggest that MRgFUS-related network reorganization may manifest differently across modalities. Structural networks may display measurable improvements in absolute clustering and efficiency, whereas functional networks reflect relative alterations in small-world organization as indexed by normalized metrics. Notably, the lack of correlation of both σ and γ with clinical tremor scores implies that while global topological reorganization may accompany treatment, it is not linked to symptom improvement.

We used modularity to evaluate the degree of segregation in functional networks—the extent to which a network can be partitioned into densely interconnected but sparsely cross-linked modules. Higher modularity indicates a more segregated system, with stronger communication within modules and weaker interactions between modules [31]. Our analysis revealed no significant differences in modularity between baseline and six months after MRgFUS, indicating a relative preservation of the large-scale modular architecture at follow-up. Interestingly, a previous DBS study reported a decrease in modularity after stimulation, consistent with enhanced cross-system integration [42]. In contrast, our results suggest that MRgFUS does not substantially disrupt modular segregation at the whole-brain level. The observed differences between DBS in the literature and our MRgFUS data highlight that distinct interventions may have different effects on modularity, which warrants further investigation. Moreover, we identified six distinct modules encompassing the temporal, the frontoparietal, the frontotemporal-insular, the subcortical, the parieto-occipital-cerebellar, and the frontotemporal-parietal modules. These modules corresponded well with several well-known functional systems: the temporal module largely overlapped with auditory and limbic networks [43], the frontoparietal with the sensorimotor network (SMN) [43], the frontotemporal-insular with the salience network [44], the subcortical with the thalamic network [45], the parieto-occipital-cerebellar with the visual-cerebellar network [46,47], and the frontotemporal-parietal with the default mode network (DMN) [46]. This alignment with well-established networks supports the neurobiological validity of our modular parcellation, providing a meaningful framework for interpreting network reorganization after MRgFUS.

At the cortical-subcortical level, we observed a trend toward increased functional connectivity between the SMN (the frontoparietal module) and the thalamic network (the subcortical module). This observation aligns with the central role of the CTC network in the pathophysiology and treatment of ET [9]. Previous studies have demonstrated aberrant connectivity within the CTC network in ET, and several lines of evidence indicate that MRgFUS modulates this circuitry. For example, a previous study from our group reported a significant reduction in excitatory drive from the cerebellum to the contralateral sensorimotor cortex, accompanied by an increased excitatory input from the primary motor cortex to cerebellar lobules V and VIII at one month post-MRgFUS [25]. Other work revealed a significant increase in functional connectivity between the left thalamus and the caudal dorsal premotor cortex three months after MRgFUS [48], while Lin et al. [20] observed long-term enhancement of a corticostriatal subnetwork with recovery of network degree, global efficiency, and transitivity. Taken together, our findings are consistent with these reports and indicate that MRgFUS may promote reorganization of cortical-subcortical interactions within the CTC network, which is the ‘oscillating central network’ of ET [9].

At the large-scale cortical level, we also noted a trend-level increase in functional connectivity between the SMN and the DMN (the frontotemporal-parietal module). The DMN is a task-negative network that is typically activated during rest and deactivated during externally oriented tasks [49], and plays an important role in cognitive processing both in normal aging and in neurodegenerative disorders [[50], [51], [52]]. Previous studies have demonstrated abnormal functional connectivity within the DMN of ET patients, suggesting that DMN dysfunction may contribute to disease manifestation [53,54]. Using ordinal trends canonical variates analysis, Xiong et al. [55] identified an ET-related fALFF pattern (ETRP-fALFF) characterized by a positive contribution of the SMN and a negative contribution of the DMN. After MRgFUS thalamotomy, spontaneous neural activity increased in the SMN but decreased in the DMN, leading the authors to propose that disruption of the DMN-SMN connectivity may be central to tremor generation, and that MRgFUS may re-establish this relationship to suppress symptoms [55]. Our results provide weak but convergent evidence in support of this interpretation: the trend-level enhancement of SMN-DMN connectivity observed in our cohort suggests that MRgFUS may also promote a broader reorganization of large-scale cortical networks.

Within the modules exhibiting higher inter-modular connectivity, we observed significant increases in betweenness centrality in three cortical hubs: the left superior frontal gyrus, the right superior parietal lobule, and the left postcentral gyrus. The left superior frontal gyrus, belonging to the frontotemporal-parietal module, has been implicated in attentional control and executive regulation [56]. Both the right superior parietal lobule and the left postcentral gyrus are part of the frontoparietal module and serve as key nodes for somatosensory-motor integration [57,58]. The observed increases in betweenness centrality indicate that these regions play a more prominent intermediary role in information transfer across the network. Notably, the betweenness centrality of the right superior parietal lobule was positively correlated with enhanced connectivity between the frontoparietal and subcortical modules, underscoring its role as a pivotal cortical relay in reestablishing cortico-thalamic communication. Together, these findings suggest that MRgFUS may not only promote reorganization of cortico-subcortical and large-scale cortical networks, but also increase the centrality and bridging role of specific cortical hubs, indicating a redistribution of information flow across modules within the CTC network and beyond.

At the whole-brain level, ET is increasingly viewed as a network disorder, but prior graph-theoretical studies report heterogeneous disruptions [11,14,15]. For example, Benito-León et al. [11] found alterations in σ and γ in ET patients, yet several global metrics only reached statistical significance under restricted threshold ranges, suggesting relatively modest large-scale network perturbations. As the present study did not include a healthy control group, we were unable to determine the extent of baseline global network abnormalities. Our findings do not suggest major global functional topological changes after MRgFUS. This aligns with a longitudinal study reporting only transient postoperative increases in global network measures, which revert toward baseline within six months, suggesting that a focal VIM lesion does not cause sustained global reorganization [20]. Moreover, the lack of significant correlations between network changes and tremor improvement may limit direct clinical implications. Tremor in ET is primarily linked to abnormalities within the CTC network [9], and alterations within this network are more closely associated with tremor severity. Indeed, one previous study focusing on a motor-tremor subnetwork has demonstrated postoperative reductions in graph-theoretical measures that correlate with clinical outcomes [18]. In contrast, the global metrics used in the present study, such as σ and γ [31], reflect whole-brain functional reorganization rather than symptom-specific network alterations, and thus may not directly capture tremor-related changes at the global level.

Our findings should be treated cautiously, as several limitations have to be acknowledged. First, the modest sample size (n = 37) reduces statistical power for node-level analyses and clinical-imaging correlations, which may limit generalizability. Second, the absence of an independent healthy or untreated ET control group precludes definitive causal attribution of the observed network changes to MRgFUS, as test-retest effects, scanner-related variability, or natural disease progression cannot be fully excluded. This limitation is particularly relevant given the modest magnitude of the observed network alterations. Third, our network construction relied on binary thresholding and community detection at a single density (0.15); although topological metrics were summarized across a sparsity range using AUC, binarization and single-density modular analysis may omit weight information and be sensitive to threshold choice. Fourth, global metrics were tested with one-tailed statistics based on an a priori hypothesis and were not corrected for multiple comparisons, which may increase the risk of Type I error [59,60]. Finally, only baseline and 6-month scans were available, precluding characterization of early and late postoperative network dynamics. Network changes may be temporally dynamic, and the 6-month time point may capture a relatively neutral phase.

In summary, MRgFUS for ET may induce selective functional network reorganization, including increased small-worldness and enhanced betweenness centrality in frontal and parietal hubs, supporting previous adaptations of the structural brain network in ET post-MRgFUS. Modular organization remained largely stable after MRgFUS, but trend-level changes in inter-modular connectivity suggest reconfiguration within the cerebello-thalamo-cortical network and large-scale cortical systems. These findings provide a better understanding of the functional reorganization underlying MRgFUS therapy. Future studies with larger cohorts, matched controls, and multi-timepoint longitudinal imaging are necessitated to confirm the robustness, temporal dynamics, and clinical relevance of these network changes.

## Ethical standards

The study was approved by the local ethics board of the University Hospital Bonn (No. 314/18), with informed consent from all participants obtained in accordance with the Declaration of Helsinki.

## Author contributions

Jinlong Liu: Writing – original draft, Methodology, Investigation, Conceptualization. Jonas Krauss: Writing – review & editing, Data Curation. Veronika Purrer: Writing – review & editing, Data Curation. Valeri Borger: Writing – review & editing, Data Curation. Markus Essler: Writing – review & editing. Alexander Radbruch: Writing – review & editing. Ullrich Wüllner: Writing – review & editing, Funding Acquisition. Neeraj Upadhyay: Writing – review & editing, Methodology, Supervision. Henning Boecker: Writing – review & editing, Funding Acquisition, Supervision.

## Declaration of competing interest

The authors declare that they have no known competing financial interests or personal relationships that could have appeared to influence the work reported in this paper.
