# Supplementary material for: Functional network reorganization following VIM-MRgFUS for essential tremor
Source: Neurotherapeutics. 2026 Feb 27;23(2):e00864. doi: 10.1016/j.neurot.2026.e00864 (PMC12993401; doi:10.1016/j.neurot.2026.e00864)
Supplement: Multimedia component 1 [file mmc1.docx]

| **Supplementary table 1** | | | | |
| --- | --- | --- | --- | --- |
| Changes in the AUC of nodal topological properties between baseline and 6 months | | | | |
| Region | Node ID ^a^ | *p* value ^b^ | | |
|  |  | Betweeness centrality | Degree centrality | Nodal efficiency |
| Superior frontal gyrus(SFG) | SFG_L_7_1 | 0.953 | 0.982 | 0.974 |
|  | SFG_R_7_1 | 0.941 | 0.982 | 0.974 |
|  | SFG_L_7_2 | 0.962 | 0.991 | 0.982 |
|  | SFG_R_7_2 | 0.941 | 0.982 | 0.974 |
|  | SFG_L_7_3 | 0.552 | 0.982 | 0.974 |
|  | SFG_R_7_3 | 0.962 | 0.985 | 0.974 |
|  | SFG_L_7_4 | 0.941 | 0.982 | 0.974 |
|  | SFG_R_7_4 | 0.999 | 0.988 | 0.974 |
|  | SFG_L_7_5 | 0.962 | 0.982 | 0.974 |
|  | SFG_R_7_5 | 0.941 | 0.982 | 0.974 |
|  | SFG_L_7_6 | 0.941 | 0.982 | 0.974 |
|  | SFG_R_7_6 | 0.941 | 0.982 | 0.974 |
|  | SFG_L_7_7 | 0.962 | 0.991 | 0.982 |
|  | SFG_R_7_7 | 0.999 | 0.982 | 0.974 |
| Middle frontal gyrus(MFG) | MFG_L_7_1 | 0.970 | 0.982 | 0.974 |
|  | MFG_R_7_1 | 0.973 | 0.982 | 0.974 |
|  | MFG_L_7_2 | 0.962 | 0.991 | 0.982 |
|  | MFG_R_7_2 | 0.973 | 0.982 | 0.974 |
|  | MFG_L_7_3 | 0.941 | 0.982 | 0.974 |
|  | MFG_R_7_3 | 0.973 | 0.991 | 0.997 |
|  | MFG_L_7_4 | 0.962 | 0.982 | 0.974 |
|  | MFG_R_7_4 | 0.941 | 0.982 | 0.974 |
|  | MFG_L_7_5 | 0.981 | 0.982 | 0.974 |
|  | MFG_R_7_5 | 0.792 | 0.982 | 0.974 |
|  | MFG_L_7_6 | 0.953 | 0.982 | 0.974 |
|  | MFG_R_7_6 | 0.962 | 0.988 | 0.974 |
|  | MFG_L_7_7 | 0.999 | 0.982 | 0.974 |
|  | MFG_R_7_7 | 0.973 | 0.991 | 0.997 |
| Inferior frontal gyrus(IFG) | IFG_L_6_1 | 0.979 | 0.982 | 0.974 |
|  | IFG_R_6_1 | 0.941 | 0.982 | 0.974 |
|  | IFG_L_6_2 | 0.973 | 0.982 | 0.974 |
|  | IFG_R_6_2 | 0.973 | 0.982 | 0.974 |
|  | IFG_L_6_3 | 0.941 | 0.982 | 0.974 |
|  | IFG_R_6_3 | 0.973 | 0.982 | 0.974 |
|  | IFG_L_6_4 | 0.953 | 0.982 | 0.974 |
|  | IFG_R_6_4 | 0.962 | 0.982 | 0.974 |
|  | IFG_L_6_5 | 0.999 | 0.985 | 0.974 |
|  | IFG_R_6_5 | 0.962 | 0.982 | 0.974 |
|  | IFG_L_6_6 | 0.953 | 0.982 | 0.974 |
|  | IFG_R_6_6 | 0.983 | 0.982 | 0.974 |
| Orbital gyrus(OrG) | OrG_L_6_1 | 0.962 | 0.982 | 0.974 |
|  | OrG_R_6_1 | 0.953 | 0.982 | 0.974 |
|  | OrG_L_6_2 | 0.970 | 0.982 | 0.974 |
|  | OrG_R_6_2 | 0.792 | 0.982 | 0.974 |
|  | OrG_L_6_3 | 0.941 | 0.982 | 0.974 |
|  | OrG_R_6_3 | 0.941 | 0.988 | 0.982 |
|  | OrG_L_6_4 | 0.973 | 0.982 | 0.974 |
|  | OrG_R_6_4 | 0.973 | 0.988 | 0.982 |
|  | OrG_L_6_5 | 0.941 | 0.988 | 0.974 |
|  | OrG_R_6_5 | 0.941 | 0.982 | 0.974 |
|  | OrG_L_6_6 | 0.962 | 0.982 | 0.974 |
|  | OrG_R_6_6 | 0.973 | 0.982 | 0.974 |
| Precentral gyrus(PrG) | PrG_L_6_1 | 0.941 | 0.988 | 0.974 |
|  | PrG_R_6_1 | 0.941 | 0.994 | 0.997 |
|  | PrG_L_6_2 | 0.941 | 0.982 | 0.974 |
|  | PrG_R_6_2 | 0.999 | 0.982 | 0.974 |
|  | PrG_L_6_3 | 0.941 | 0.982 | 0.974 |
|  | PrG_R_6_3 | 0.962 | 0.982 | 0.974 |
|  | PrG_L_6_4 | 0.941 | 0.991 | 0.997 |
|  | PrG_R_6_4 | 0.941 | 0.982 | 0.974 |
|  | PrG_L_6_5 | 0.953 | 0.982 | 0.974 |
|  | PrG_R_6_5 | 0.941 | 0.982 | 0.974 |
|  | PrG_L_6_6 | 0.962 | 0.982 | 0.974 |
|  | PrG_R_6_6 | 0.962 | 0.982 | 0.974 |
| PCL, Paracentral lobule | PCL_L_2_1 | 0.973 | 0.988 | 0.982 |
|  | PCL_R_2_1 | 0.941 | 0.991 | 0.997 |
|  | PCL_L_2_2 | 0.962 | 0.982 | 0.974 |
|  | PCL_R_2_2 | 0.962 | 0.982 | 0.974 |
| Superior temporal gyrus(STG) | STG_L_6_1 | 0.792 | 0.982 | 0.974 |
|  | STG_R_6_1 | 0.973 | 0.991 | 0.982 |
|  | STG_L_6_2 | 0.953 | 0.982 | 0.974 |
|  | STG_R_6_2 | 0.941 | 0.982 | 0.974 |
|  | STG_L_6_3 | 0.941 | 0.982 | 0.974 |
|  | STG_R_6_3 | 0.941 | 0.982 | 0.974 |
|  | STG_L_6_4 | 0.997 | 0.988 | 0.974 |
|  | STG_R_6_4 | 0.962 | 0.982 | 0.974 |
|  | STG_L_6_5 | 0.941 | 0.982 | 0.974 |
|  | STG_R_6_5 | 0.962 | 0.988 | 0.974 |
|  | STG_L_6_6 | 0.999 | 0.982 | 0.974 |
|  | STG_R_6_6 | 0.941 | 0.988 | 0.974 |
| Middle temporal gyrus(MTG) | MTG_L_4_1 | 0.999 | 0.988 | 0.974 |
|  | MTG_R_4_1 | 0.941 | 0.982 | 0.974 |
|  | MTG_L_4_2 | 0.953 | 0.982 | 0.974 |
|  | MTG_R_4_2 | 0.962 | 0.982 | 0.974 |
|  | MTG_L_4_3 | 0.941 | 0.982 | 0.974 |
|  | MTG_R_4_3 | 0.962 | 0.985 | 0.974 |
|  | MTG_L_4_4 | 0.953 | 0.988 | 0.979 |
|  | MTG_R_4_4 | 0.962 | 0.982 | 0.974 |
| Inferior temporal gyrus(ITG) | ITG_L_7_1 | 0.941 | 0.982 | 0.974 |
|  | ITG_R_7_1 | 0.941 | 0.982 | 0.974 |
|  | ITG_L_7_2 | 0.941 | 0.982 | 0.974 |
|  | ITG_R_7_2 | 0.941 | 0.982 | 0.974 |
|  | ITG_L_7_3 | 0.953 | 0.982 | 0.974 |
|  | ITG_R_7_3 | 0.973 | 0.982 | 0.974 |
|  | ITG_L_7_4 | 0.941 | 0.982 | 0.974 |
|  | ITG_R_7_4 | 0.973 | 0.982 | 0.974 |
|  | ITG_L_7_5 | 0.941 | 0.982 | 0.974 |
|  | ITG_R_7_5 | 0.990 | 0.988 | 0.982 |
|  | ITG_L_7_6 | 0.941 | 0.988 | 0.982 |
|  | ITG_R_7_6 | 0.751 | 0.982 | 0.974 |
|  | ITG_L_7_7 | 0.990 | 0.982 | 0.974 |
|  | ITG_R_7_7 | 0.941 | 0.988 | 0.974 |
| Fusiform gyrus(FuG) | FuG_L_3_1 | 0.999 | 0.982 | 0.974 |
|  | FuG_R_3_1 | 0.962 | 0.982 | 0.974 |
|  | FuG_L_3_2 | 0.973 | 0.982 | 0.974 |
|  | FuG_R_3_2 | 0.973 | 0.982 | 0.974 |
|  | FuG_L_3_3 | 0.941 | 0.982 | 0.974 |
|  | FuG_R_3_3 | 0.962 | 0.982 | 0.974 |
| Parahippocampal gyrus(PhG) | PhG_L_6_1 | 0.941 | 0.982 | 0.974 |
|  | PhG_R_6_1 | 0.941 | 0.982 | 0.974 |
|  | PhG_L_6_2 | 0.990 | 0.982 | 0.974 |
|  | PhG_R_6_2 | 0.973 | 0.991 | 0.974 |
|  | PhG_L_6_3 | 0.941 | 0.982 | 0.974 |
|  | PhG_R_6_3 | 0.999 | 0.982 | 0.974 |
|  | PhG_L_6_4 | 0.990 | 0.991 | 0.987 |
|  | PhG_R_6_4 | 0.962 | 0.982 | 0.974 |
|  | PhG_L_6_5 | 0.973 | 0.991 | 0.997 |
|  | PhG_R_6_5 | 0.973 | 0.982 | 0.974 |
|  | PhG_L_6_6 | 0.962 | 0.982 | 0.974 |
|  | PhG_R_6_6 | 0.973 | 0.985 | 0.974 |
| Posterior superior temporal sulcus(pSTS) | pSTS_L_2_1 | 0.941 | 0.982 | 0.974 |
|  | pSTS_R_2_1 | 0.941 | 0.982 | 0.974 |
|  | pSTS_L_2_2 | 0.941 | 0.982 | 0.974 |
|  | pSTS_R_2_2 | 0.941 | 0.982 | 0.974 |
| Superior parietal lobule(SPL) | SPL_L_5_1 | 0.656 | 0.982 | 0.974 |
|  | SPL_R_5_1 | 0.552 | 0.982 | 0.974 |
|  | SPL_L_5_2 | 0.962 | 0.982 | 0.974 |
|  | SPL_R_5_2 | 0.941 | 0.982 | 0.974 |
|  | SPL_L_5_3 | 0.973 | 0.982 | 0.974 |
|  | SPL_R_5_3 | 0.751 | 0.982 | 0.974 |
|  | SPL_L_5_4 | 0.941 | 0.982 | 0.974 |
|  | SPL_R_5_4 | 0.973 | 0.988 | 0.982 |
|  | SPL_L_5_5 | 0.941 | 0.982 | 0.974 |
|  | SPL_R_5_5 | 0.962 | 0.982 | 0.974 |
| Inferior parietal lobule(IPL) | IPL_L_6_1 | 0.962 | 0.982 | 0.974 |
|  | IPL_R_6_1 | 0.962 | 0.982 | 0.974 |
|  | IPL_L_6_2 | 0.962 | 0.991 | 0.982 |
|  | IPL_R_6_2 | 0.941 | 0.982 | 0.974 |
|  | IPL_L_6_3 | 0.999 | 0.988 | 0.997 |
|  | IPL_R_6_3 | 0.973 | 0.982 | 0.974 |
|  | IPL_L_6_4 | 0.941 | 0.982 | 0.974 |
|  | IPL_R_6_4 | 0.962 | 0.982 | 0.974 |
|  | IPL_L_6_5 | 0.962 | 0.982 | 0.974 |
|  | IPL_R_6_5 | 0.973 | 0.991 | 0.997 |
|  | IPL_L_6_6 | 0.973 | 0.982 | 0.974 |
|  | IPL_R_6_6 | 0.941 | 0.985 | 0.982 |
| Precuneus(Pcun) | PCun_L_4_1 | 0.751 | 0.982 | 0.974 |
|  | PCun_R_4_1 | 0.941 | 0.982 | 0.974 |
|  | PCun_L_4_2 | 0.973 | 0.985 | 0.982 |
|  | PCun_R_4_2 | 0.962 | 0.982 | 0.974 |
|  | PCun_L_4_3 | 0.953 | 0.982 | 0.974 |
|  | PCun_R_4_3 | 0.941 | 0.982 | 0.974 |
|  | PCun_L_4_4 | 0.973 | 0.982 | 0.974 |
|  | PCun_R_4_4 | 0.962 | 0.982 | 0.974 |
| Postcentral gyrus(PoG) | PoG_L_4_1 | 0.751 | 0.982 | 0.974 |
|  | PoG_R_4_1 | 0.941 | 0.982 | 0.974 |
|  | PoG_L_4_2 | 0.941 | 0.985 | 0.974 |
|  | PoG_R_4_2 | 0.973 | 0.982 | 0.974 |
|  | PoG_L_4_3 | 0.970 | 0.988 | 0.974 |
|  | PoG_R_4_3 | 0.941 | 0.988 | 0.974 |
|  | PoG_L_4_4 | 0.751 | 0.982 | 0.974 |
|  | PoG_R_4_4 | 0.941 | 0.982 | 0.974 |
| Insular gyrus(INS) | INS_L_6_1 | 0.941 | 0.982 | 0.974 |
|  | INS_R_6_1 | 0.973 | 0.982 | 0.974 |
|  | INS_L_6_2 | 0.973 | 0.991 | 0.987 |
|  | INS_R_6_2 | 0.973 | 0.982 | 0.974 |
|  | INS_L_6_3 | 0.973 | 0.982 | 0.974 |
|  | INS_R_6_3 | 0.990 | 0.982 | 0.974 |
|  | INS_L_6_4 | 0.962 | 0.982 | 0.974 |
|  | INS_R_6_4 | 0.962 | 0.982 | 0.974 |
|  | INS_L_6_5 | 0.953 | 0.982 | 0.974 |
|  | INS_R_6_5 | 0.962 | 0.982 | 0.974 |
|  | INS_L_6_6 | 0.941 | 0.988 | 0.982 |
|  | INS_R_6_6 | 0.962 | 0.988 | 0.974 |
| Cingulate gyrus(CG) | CG_L_7_1 | 0.999 | 0.982 | 0.974 |
|  | CG_R_7_1 | 0.941 | 0.988 | 0.974 |
|  | CG_L_7_2 | 0.962 | 0.982 | 0.974 |
|  | CG_R_7_2 | 0.941 | 0.982 | 0.974 |
|  | CG_L_7_3 | 0.997 | 0.982 | 0.974 |
|  | CG_R_7_3 | 0.941 | 0.982 | 0.974 |
|  | CG_L_7_4 | 0.941 | 0.982 | 0.974 |
|  | CG_R_7_4 | 0.894 | 0.982 | 0.974 |
|  | CG_L_7_5 | 0.962 | 0.982 | 0.974 |
|  | CG_R_7_5 | 0.941 | 0.982 | 0.974 |
|  | CG_L_7_6 | 0.973 | 0.988 | 0.974 |
|  | CG_R_7_6 | 0.962 | 0.982 | 0.974 |
|  | CG_L_7_7 | 0.962 | 0.991 | 0.977 |
|  | CG_R_7_7 | 0.997 | 0.982 | 0.974 |
| MedioVentral occipital cortex(MVOcC) | MVOcC_L_5_1 | 0.962 | 0.982 | 0.974 |
|  | MVOcC_R_5_1 | 0.962 | 0.982 | 0.974 |
|  | MVOcC_L_5_2 | 0.973 | 0.982 | 0.974 |
|  | MVOcC_R_5_2 | 0.973 | 0.982 | 0.974 |
|  | MVOcC_L_5_3 | 0.999 | 0.982 | 0.974 |
|  | MVOcC_R_5_3 | 0.941 | 0.982 | 0.974 |
|  | MVOcC_L_5_4 | 0.962 | 0.982 | 0.974 |
|  | MVOcC_R_5_4 | 0.941 | 0.982 | 0.974 |
|  | MVOcC_L_5_5 | 0.999 | 0.982 | 0.974 |
|  | MVOcC_R_5_5 | 0.941 | 0.982 | 0.974 |
| Lateral occipital cortex(LOcC) | LOcC_L_4_1 | 0.973 | 0.982 | 0.974 |
|  | LOcC_R_4_1 | 0.792 | 0.982 | 0.974 |
|  | LOcC_L_4_2 | 0.999 | 0.982 | 0.974 |
|  | LOcC_R_4_2 | 0.941 | 0.988 | 0.982 |
|  | LOcC_L_4_3 | 0.990 | 0.982 | 0.974 |
|  | LOcC_R_4_3 | 0.962 | 0.982 | 0.974 |
|  | LOcC_L_4_4 | 0.970 | 0.982 | 0.974 |
|  | LOcC_R_4_4 | 0.962 | 0.982 | 0.974 |
|  | LOcC_L_2_1 | 0.953 | 0.982 | 0.974 |
|  | LOcC_R_2_1 | 0.941 | 0.982 | 0.974 |
|  | LOcC_L_2_2 | 0.941 | 0.982 | 0.974 |
|  | LOcC_R_2_2 | 0.990 | 0.982 | 0.974 |
| Amygdala(Amyg) | Amyg_L_2_1 | 0.941 | 0.982 | 0.974 |
|  | Amyg_R_2_1 | 0.953 | 0.982 | 0.974 |
|  | Amyg_L_2_2 | 0.962 | 0.982 | 0.974 |
|  | Amyg_R_2_2 | 0.973 | 0.982 | 0.974 |
| Hippocampus(Hipp) | Hipp_L_2_1 | 0.941 | 0.982 | 0.974 |
|  | Hipp_R_2_1 | 0.990 | 0.982 | 0.974 |
|  | Hipp_L_2_2 | 0.990 | 0.982 | 0.974 |
|  | Hipp_R_2_2 | 0.962 | 0.982 | 0.974 |
| Basal ganglia(BG) | BG_L_6_1 | 0.997 | 0.994 | 0.982 |
|  | BG_R_6_1 | 0.973 | 0.982 | 0.974 |
|  | BG_L_6_2 | 0.792 | 0.982 | 0.974 |
|  | BG_R_6_2 | 0.941 | 0.982 | 0.974 |
|  | BG_L_6_3 | 0.962 | 0.982 | 0.974 |
|  | BG_R_6_3 | 0.990 | 0.982 | 0.974 |
|  | BG_L_6_4 | 0.962 | 0.982 | 0.974 |
|  | BG_R_6_4 | 0.973 | 0.982 | 0.974 |
|  | BG_L_6_5 | 0.973 | 0.988 | 0.974 |
|  | BG_R_6_5 | 0.941 | 0.982 | 0.974 |
|  | BG_L_6_6 | 0.990 | 0.982 | 0.997 |
|  | BG_R_6_6 | 0.973 | 0.982 | 0.974 |
| Thalamus(Tha) | Tha_L_8_1 | 0.941 | 0.982 | 0.974 |
|  | Tha_R_8_1 | 0.845 | 0.982 | 0.974 |
|  | Tha_L_8_2 | 0.941 | 0.982 | 0.974 |
|  | Tha_R_8_2 | 0.973 | 0.988 | 0.974 |
|  | Tha_L_8_3 | 0.973 | 0.982 | 0.974 |
|  | Tha_R_8_3 | 0.941 | 0.982 | 0.974 |
|  | Tha_L_8_4 | 0.962 | 0.988 | 0.974 |
|  | Tha_R_8_4 | 0.941 | 0.982 | 0.974 |
|  | Tha_L_8_5 | 0.970 | 0.982 | 0.974 |
|  | Tha_R_8_5 | 0.962 | 0.982 | 0.974 |
|  | Tha_L_8_6 | 0.962 | 0.982 | 0.974 |
|  | Tha_R_8_6 | 0.953 | 0.982 | 0.974 |
|  | Tha_L_8_7 | 0.973 | 0.982 | 0.974 |
|  | Tha_R_8_7 | 0.953 | 0.982 | 0.974 |
|  | Tha_L_8_8 | 0.941 | 0.982 | 0.974 |
|  | Tha_R_8_8 | 0.990 | 0.982 | 0.974 |
| Cerebellum(CB) | CB_L_I-IV | 0.962 | 0.982 | 0.974 |
|  | CB_R_I-IV | 0.973 | 0.982 | 0.974 |
|  | CB_L_V | 0.962 | 0.985 | 0.974 |
|  | CB_R_V | 0.962 | 0.982 | 0.974 |
|  | CB_L_VI | 0.941 | 0.982 | 0.974 |
|  | CB_V_VI | 0.973 | 0.982 | 0.974 |
|  | CB_R_VI | 0.962 | 0.982 | 0.974 |
|  | CB_L_Crus_I | 0.990 | 0.982 | 0.974 |
|  | CB_R_Crus_I | 0.953 | 0.988 | 0.982 |
|  | CB_L_Crus_II | 0.941 | 0.982 | 0.974 |
|  | CB_V_Crus_II | 0.999 | 0.994 | 0.974 |
|  | CB_R_Crus_II | 0.792 | 0.982 | 0.974 |
|  | CB_L_VIIb | 0.941 | 0.982 | 0.974 |
|  | CB_V_VIIb | 0.953 | 0.988 | 0.982 |
|  | CB_R_VIIb | 0.990 | 0.988 | 0.974 |
|  | CB_L_VIIIa | 0.962 | 0.994 | 0.982 |
|  | CB_V_VIIIa | 0.953 | 0.982 | 0.974 |
|  | CB_R_VIIIa | 0.962 | 0.994 | 0.982 |
|  | CB_L_VIIIb | 0.962 | 0.985 | 0.974 |
|  | CB_V_VIIIb | 0.973 | 0.988 | 0.982 |
|  | CB_R_VIIIb | 0.953 | 0.982 | 0.974 |
|  | CB_L_IX | 0.954 | 0.982 | 0.974 |
|  | CB_V_IX | 0.999 | 0.982 | 0.974 |
|  | CB_R_IX | 0.970 | 0.982 | 0.974 |
|  | CB_L_X | 0.962 | 0.982 | 0.974 |
|  | CB_V_X | 0.962 | 0.982 | 0.974 |
|  | CB_R_X | 0.941 | 0.982 | 0.974 |
| L, left; R, right; V, vermis.  ^a^ All the nodes are from the Human Brainnetome Atlas.  ^b^ The FDR correction was applied to local topological properties. | | | | |

| **Supplementary table 2** | | | | | |
| --- | --- | --- | --- | --- | --- |
| Changes in nodal topological properties within module 2, 4 and 6 between baseline and 6 months | | | | | |
| Region | Node ID ^a^ | *p* value ^b^ | | | |
|  |  | Participation coefficient | Degree centrality | Betweenness centrality | Nodal efficiency |
| Superior frontal gyrus(SFG) | SFG_R_7_7 | 0.964 | 0.957 | 0.947 | 0.937 |
|  | SFG_R_7_5 | 0.797 | 0.957 | 0.947 | 0.937 |
|  | SFG_R_7_4 | 0.650 | 0.959 | 0.817 | 0.937 |
|  | SFG_R_7_3 | 0.874 | 0.966 | 0.947 | 0.937 |
|  | SFG_R_7_2 | 0.797 | 0.957 | 0.462 | 0.937 |
|  | SFG_L_7_7 | 0.874 | 0.959 | 0.982 | 0.974 |
|  | SFG_L_7_5 | 0.892 | 0.957 | 0.817 | 0.937 |
|  | SFG_L_7_4 | 0.810 | 0.959 | 0.817 | 0.937 |
|  | **SFG_L_7_3** | 0.714 | 0.957 | **0.036*** | 0.937 |
|  | SFG_L_7_2 | 0.968 | 0.959 | 0.947 | 0.937 |
| Superior parietal lobule(SPL) | SPL_R_5_4 | 0.810 | 0.959 | 0.953 | 0.937 |
|  | **SPL_R_5_3** | 0.765 | 0.898 | **0.036*** | 0.937 |
|  | SPL_R_5_1 | 0.397 | 0.898 | 0.355 | 0.937 |
|  | SPL_L_5_4 | 0.797 | 0.957 | 0.947 | 0.937 |
|  | SPL_L_5_3 | 0.397 | 0.957 | 0.947 | 0.937 |
|  | SPL_L_5_1 | 0.207 | 0.957 | 0.402 | 0.937 |
| Orbital gyrus(OrG) | OrG_R_6_5 | 0.797 | 0.957 | 0.817 | 0.937 |
|  | OrG_R_6_4 | 0.879 | 0.959 | 0.947 | 0.937 |
|  | OrG_R_6_3 | 0.818 | 0.986 | 0.947 | 0.974 |
|  | OrG_R_6_2 | 0.874 | 0.957 | 0.596 | 0.937 |
|  | OrG_R_6_1 | 0.797 | 0.959 | 0.723 | 0.937 |
|  | OrG_L_6_5 | 0.892 | 0.981 | 0.947 | 0.937 |
|  | OrG_L_6_4 | 0.351 | 0.994 | 0.947 | 0.937 |
|  | OrG_L_6_3 | 0.888 | 0.957 | 0.817 | 0.937 |
|  | OrG_L_6_2 | 0.918 | 0.957 | 0.947 | 0.937 |
|  | OrG_L_6_1 | 0.714 | 0.957 | 0.817 | 0.937 |
| Paracentral lobule(PCL) | PCL_R_2_2 | 0.767 | 0.959 | 0.929 | 0.937 |
|  | PCL_R_2_1 | 0.841 | 0.959 | 0.434 | 0.937 |
|  | PCL_L_2_2 | 0.981 | 0.959 | 0.963 | 0.937 |
|  | PCL_L_2_1 | 0.927 | 0.975 | 0.947 | 0.937 |
| Precentral gyrus(PrG) | PrG_R_6_4 | 0.878 | 0.898 | 0.628 | 0.937 |
|  | PrG_R_6_3 | 0.810 | 0.957 | 0.947 | 0.937 |
|  | PrG_R_6_2 | 0.714 | 0.959 | 0.947 | 0.974 |
|  | PrG_L_6_4 | 0.918 | 0.959 | 0.434 | 0.937 |
|  | PrG_L_6_3 | 0.714 | 0.959 | 0.947 | 0.937 |
|  | PrG_L_6_2 | 0.918 | 0.959 | 0.817 | 0.937 |
|  | PrG_L_6_1 | 0.797 | 0.959 | 0.758 | 0.937 |
| Postcentral gyrus(PoG) | PoG_R_4_4 | 0.714 | 0.959 | 0.723 | 0.937 |
|  | PoG_R_4_3 | 0.797 | 0.957 | 0.689 | 0.937 |
|  | PoG_R_4_1 | 0.734 | 0.959 | 0.758 | 0.937 |
|  | PoG_L_4_4 | 0.797 | 0.957 | 0.593 | 0.937 |
|  | PoG_L_4_3 | 0.714 | 0.959 | 0.963 | 0.937 |
|  | **PoG_L_4_1** | 0.797 | 0.959 | **0.045*** | 0.937 |
| Precuneus(Pcun) | PCun_R_4_4 | 0.765 | 0.959 | 0.947 | 0.974 |
|  | PCun_R_4_2 | 0.841 | 0.959 | 0.947 | 0.937 |
|  | PCun_L_4_4 | 0.818 | 0.959 | 0.963 | 0.937 |
|  | PCun_L_4_3 | 0.918 | 0.957 | 0.817 | 0.937 |
|  | PCun_L_4_2 | 0.810 | 0.957 | 0.947 | 0.937 |
| Middle temporal gyrus(MTG) | MTG_R_4_4 | 0.714 | 0.957 | 0.817 | 0.937 |
|  | MTG_R_4_1 | 0.249 | 0.959 | 0.947 | 0.937 |
|  | MTG_L_4_4 | 0.767 | 0.959 | 0.947 | 0.937 |
|  | MTG_L_4_2 | 0.859 | 1.000 | 0.817 | 0.937 |
|  | MTG_L_4_1 | 0.207 | 0.959 | 0.817 | 0.937 |
|  | MFG_R_7_7 | 0.734 | 0.957 | 0.963 | 0.937 |
|  | MFG_R_7_5 | 0.810 | 0.957 | 0.723 | 0.937 |
|  | MFG_L_7_7 | 0.879 | 0.957 | 0.948 | 0.937 |
|  | MFG_L_7_6 | 0.734 | 0.966 | 0.817 | 0.937 |
|  | MFG_L_7_5 | 0.927 | 0.959 | 0.947 | 0.937 |
|  | MFG_L_7_3 | 0.918 | 0.959 | 0.947 | 0.937 |
| Inferior temporal gyrus(ITG) | ITG_R_7_6 | 0.874 | 0.139 | 0.723 | 0.937 |
|  | ITG_L_7_6 | 0.767 | 0.959 | 0.947 | 0.937 |
| Inferior parietal lobule(IPL) | IPL_R_6_5 | 0.874 | 0.966 | 0.947 | 0.950 |
|  | IPL_R_6_3 | 0.892 | 0.957 | 0.953 | 0.937 |
|  | IPL_R_6_2 | 0.207 | 0.959 | 0.723 | 0.937 |
|  | IPL_L_6_5 | 0.734 | 0.957 | 0.947 | 0.937 |
|  | IPL_L_6_3 | 0.971 | 0.959 | 0.947 | 0.937 |
|  | IPL_L_6_2 | 0.765 | 0.959 | 0.947 | 0.937 |
| Cingulate gyrus(CG) | CG_R_7_7 | 0.767 | 0.959 | 0.947 | 0.937 |
|  | CG_R_7_4 | 0.797 | 0.959 | 0.723 | 0.937 |
|  | CG_R_7_1 | 0.797 | 0.959 | 0.947 | 0.937 |
|  | CG_L_7_7 | 0.918 | 0.959 | 0.947 | 0.937 |
|  | CG_L_7_6 | 0.734 | 0.973 | 0.947 | 0.937 |
|  | CG_L_7_4 | 0.797 | 0.959 | 0.758 | 0.937 |
|  | CG_L_7_1 | 0.874 | 0.957 | 0.947 | 0.937 |
| Basal ganglia(BG) | BG_R_6_5 | 0.810 | 0.957 | 0.771 | 0.937 |
|  | BG_R_6_3 | 0.918 | 0.959 | 0.817 | 0.990 |
|  | BG_R_6_1 | 0.714 | 0.959 | 0.982 | 0.937 |
|  | BG_L_6_5 | 0.765 | 0.959 | 0.947 | 0.937 |
|  | BG_L_6_3 | 0.918 | 0.959 | 0.947 | 0.937 |
|  | BG_L_6_1 | 0.918 | 0.957 | 0.947 | 0.937 |
| Thalamus(Tha) | Tha_R_8_8 | 0.892 | 0.957 | 0.947 | 0.937 |
|  | Tha_R_8_7 | 0.874 | 0.966 | 0.947 | 0.937 |
|  | Tha_R_8_6 | 0.812 | 0.959 | 0.758 | 0.944 |
|  | Tha_R_8_5 | 0.767 | 0.957 | 0.947 | 0.937 |
|  | Tha_R_8_4 | 0.892 | 0.966 | 0.953 | 0.974 |
|  | Tha_R_8_3 | 0.767 | 0.957 | 0.758 | 0.937 |
|  | Tha_R_8_2 | 0.892 | 0.959 | 0.947 | 0.937 |
|  | Tha_R_8_1 | 0.874 | 0.959 | 0.723 | 0.937 |
|  | Tha_L_8_8 | 0.810 | 0.959 | 0.817 | 0.937 |
|  | Tha_L_8_7 | 0.714 | 0.959 | 0.947 | 0.937 |
|  | Tha_L_8_6 | 0.207 | 0.957 | 0.723 | 0.937 |
|  | Tha_L_8_5 | 0.964 | 0.957 | 0.947 | 0.937 |
|  | Tha_L_8_4 | 0.810 | 0.986 | 0.947 | 0.937 |
|  | Tha_L_8_3 | 0.918 | 0.959 | 0.947 | 0.937 |
|  | Tha_L_8_2 | 0.697 | 0.957 | 0.596 | 0.937 |
|  | Tha_L_8_1 | 0.810 | 0.959 | 0.947 | 0.937 |
| L, left; R, right.  ^a^ All the nodes are from the Human Brainnetome Atlas.  ^b^ The FDR correction was applied to local topological properties.  * Significant differences are marked with an asterisk (**p* < 0.05). | | | | | |
